# Establishment of goat mammary organoid cultures modeling the mammary gland development and lactation

**DOI:** 10.1186/s40104-024-01084-7

**Published:** 2024-10-01

**Authors:** Lei Jia, Wenying Zhang, Tao Luo, Yongtao Li, Jianhong Shu, Julie Strand, Yuan Yue, Stig Purup, Jianxin Liu, Hengbo Shi

**Affiliations:** 1 Zhejiang Key Laboratory of Cow Genetic Improvement & Milk Quality Research, Institute of Dairy Science, College of Animal Sciences, Zhejiang University, Hangzhou, 310058 China; 2 College of Life Sciences and Medicine, Zhejiang Sci-Tech University, Hangzhou, 310018 China; 3 Department of Animal and Veterinary Sciences, Aarhus University, Tjele, DK-8830 Denmark; 4 Ministry of Education Key Laboratory of Molecular Animal Nutrition, Zhejiang University, Hangzhou, 310058 China

**Keywords:** Cell-based milk production, Lactation, Mammary gland, Organoid

## Abstract

**Background:**

Although several cell culture systems have been developed to investigate the function of the mammary gland in dairy livestock, they have potential limitations, such as the loss of alveolar structure or genetic and phenotypic differences from their native counterparts. Overcoming these challenges is crucial for lactation research. Development of protocols to establish lactating organoid of livestock represents a promising goal for the future. In this study, we developed a protocol to establish a culture system for mammary organoids in dairy goats to model the mammary gland development and lactation process.

**Results:**

The organoids cultured within an extracellular matrix gel maintained a bilayer structure that closely resembled the native architecture of mammary tissue. The expansion of mammary organoids was significantly promoted by growth factors containing epidermal growth factor and fibroblast growth factor 2 whereas the proliferative index of the organoids was significantly inhibited by the treatment with WNT inhibitors. Upon stimulation with a lactogenic medium containing prolactin, the mammary organoids exhibited efficient lactation, characterized by the accumulation of lipid droplets in the lumen space. The lactation could be sustained for more than 3 weeks. Importantly, the expression patterns of genes related to fatty acid synthesis and milk proteins in lactating organoids closely mirrored those observed in mammary tissues. These observations were confirmed by data from proteomic analysis that the bulk of milk proteins was produced in the lactating organoids.

**Conclusion:**

This study is the first to establish a mammary organoid culture system modeling the mammary gland development and lactation process in ruminants. The efficient induction of lactation in ruminant mammary organoids holds promises for advancing the field of cell-based milk bio-manufacture in the food industry.

**Supplementary Information:**

The online version contains supplementary material available at 10.1186/s40104-024-01084-7.

## Introduction

The mammary gland is an unique organ that secretes milk for the nourishment of the newborn. This secretory organ primarily undergoes postnatal development and remains highly responsive to hormonal cues [1]. Following pregnancy, the mammary epithelium undergoes extensive cell proliferation, ductal branching, and differentiation of alveoli, all of which are necessary for the development of secretory alveolar structures. The mammary epithelium is exposed to the surge of prolactin and adrenal steroids, setting the stage for its transformation into proficient secretory alveoli [2]. The mature secretory alveoli operate as efficient milk-producing factories, synthesizing essential milk components, including milk proteins [e.g*.*, beta casein (CSN2) and kappa casein (CSN3)] and milk fat. The process of weaning initiates the involution of the mammary gland, a phase in which the biosynthesis of milk gradually diminishes [3].

The mammary glands of dairy livestock serve as a vital economic organ, providing natural food for human nutrition. A comprehensive understanding of the lactation process within mammary tissue in dairy livestock is crucial for enhancing both milk yield and improving milk quality. The structural unit of mammary alveoli in the mammary gland features a bilayer architecture, comprising outer myoepithelial cells and inner luminal cells [4]. Luminal cells play a central role as secretory mammary epithelial cells, while myoepithelial cells contract to facilitate the expulsion of milk from the secretory luminal cells. In the pursuit of studying the biology of lactating mammary tissue, conventional two-dimensional (2D) cultured mammary epithelial cells have historically been employed in ruminants [5–7]. However, this approach has been associated with potential limitations, including the loss of three-dimensional alveolar structure and the emergence of genetic and phenotypic differences from their native counterparts, as highlighted in recent research [8]. A significant concern in 2D culture is the frequent loss of expression of hormone receptors in various ruminant mammary epithelial cell lines [8]. Consequently, there is a compelling need for the development of an effective in vitro model to comprehensively study the lactation biology of the mammary gland in ruminants.

The adoption of 3D-cultured models has emerged as a valuable approach to modeling the function of mammary glands in dairy livestock [9–13]. These initial 3D culture systems, characterized by the use of extracellular matrix (ECM) gel to embed mammary epithelial cells, have demonstrated the ability to form an acinus-like structure. However, most of these reports used mammary epithelial cell lines or cells from fresh milk, resulting in loss of bilayer architecture [9–12]. Mammary organoids have been established to preserve tissue-specific cell differentiation and functionality in mouse and human models [8, 14]. Compared with the initial 3D culture systems only using epithelial cells, the organoids contain a bilayer alveolar structure modeling biological processes like branching morphogenesis, polarization, and lumen formation [14, 15]. While some studies have explored the aspect of branching morphogenesis of mammary organoids, the essential function of the mammary gland, lactation, has received far less attention [8, 16, 17]. Notably, recent data from rodent models have shown that primary mammary organoids can undergo pregnancy-associated alveologenesis and milk production upon hormonal treatment [18, 19]. Nevertheless, it is essential to acknowledge that the adult mammary gland in livestock exhibits a more complex architecture, characterized by terminal ductal lobular units, which represents a more advanced state of epithelial development toward the lactating architecture compared to the mice [20, 21]. These architectural disparities between ruminants and rodents pose challenges when attempting to develop lactating mammary organoids in ruminant livestock models [8].

The objective of this study is to establish a mammary organoid culture system dedicated to the investigation of mammary gland development and lactation biology in dairy livestock. In the current study, we have firstly established a goat mammary organoid culture system. The results highlighted the potentials of these mammary organoids as a valuable platform for advancing our understanding of ruminant mammary biology.

## Materials and methods

### Tissue collection

For the preparation of primary mammary organoids, mammary tissue (parenchymal area, about 5 g) was obtained from female Saanen dairy goats aged 8 to 10 months (virgin). The tissue collection was performed following slaughter, and the mammary tissue was carefully washed with a PBS (phosphate-buffered saline) solution (C0221A, Beyotime Biotechnology Co., Ltd., Shanghai, China) under sterile laboratory conditions. Mammary tissues for morphological comparison were collected from three-year-old Saanen dairy goats at different physiological states, including peak lactation (*n* = 3 goats, 100 d postpartum) and dry period (*n* = 3 goats). Approximately 1 g of mammary tissue was collected by biopsies and a small part of tissue was subsequently fixed in a 4% paraformaldehyde solution (PFA, P0099, Beyotime Biotechnology Co., Ltd., Shanghai, China) for immunofluorescent staining.

### Isolation of goat primary mammary epithelial organoids

The isolation procedure for the digestion of collected goat mammary tissues was performed with previously established methods with some modifications [22, 23]. Briefly, the collected mammary tissue was minced into fragments and digested with 1× collagenase/hyaluronidase (10×, 3,000 U/mL collagenase and 1,000 U/mL hyaluronidase, 07912, StemCell Technologies, Cambridge, USA) in DMEM/F12 medium (SH30004.04, HyClone, Logan, USA) containing 5% fetal bovine serum (SH30084.03, Hyclone, Logan, USA). Generally, we add 10 mL digested solution per 1 g mammary tissue in 50-mL tube. This digestion mixture was incubated at 37 °C for 1.5 h with gentle shaking at 200 r/min. Erythrocytes were lysed with ammonium chloride (diluted in PBS, 0.8%, 07800, StemCell Technologies, Cambridge, USA) for 5 min. The tissue suspension was treated with 20 U/mL DNase I (Yuanye Biological Co., Ltd., Shanghai, China) for 5 min at room temperature and exposed to three rounds of differential centrifugation at 350 × *g* for 60 s, 30 s, and 10 s to discard single cells and lymphocytes. The organoids were re-suspended in 10 mL basal medium [BM; 5 mg/L insulin (I3536, Sigma, St. Louis, USA), 100 U/mL of penicillin/streptomycin (P4333, Sigma, St. Louis, USA), in DMEM/F12 (SH30004.04, HyClone, Logan, USA)] and kept on ice for 3D culture.

### 3D culture of goat mammary organoids

Before plating, thoroughly mix the organoids to ensure a homogeneous mixture. Transfer 20 μL of this mixture to a 30-mm dish (Corning, NY, USA) and use a microscope to count the number of organoids in this sample volume. This will help determine the density of the organoids. Each well was seeded with approximately 200 organoids. Take the necessary volumes of organoid suspension and remove the supernatant carefully by centrifuging at 600 × *g* for 5 min. Extracted mammary organoids were mixed with cold growth factor-reduced ECM gel (E6909, Sigma, St. Louis, USA) and plated in domes in 24-well culture plates (one dome per well, 100 µL of undiluted Matrigel per dome). After a 60-min incubation at 37 °C to allow for proper Matrigel solidification, a growth medium was gently added. This growth medium (GM) consisted of BM supplemented with growth factors containing 2.5 nmol/L FGF2, 2.5 nmol/L FGF7, 2.5 nmol/L FGF10, 50 ng/mL EGF (all from Thermo Fisher Scientific, Waltham, USA). The organoids were cultured in a CO_2_ (5%) incubator at 37 °C. To induce lactogenesis, the organoids were cultured with lactogenic medium [(LM): BM with 2 µg/mL prolactin]. The sheep prolactin (CW72, Novoprotein, Suzhou, China) and human prolactin (Pepro Tech, Rocky Hill, USA) were used to assess their role in lactation induction. The GM or LM maintaining organoids was renewed every 2 d.

The procedures for replicating organoids were as previously described [17, 18]. Briefly, 3D cultures were rinsed with cold cell recovery solution (Yeasen Biotechnology Co., Ltd., Shanghai, China) and disintegrated by pipetting up and down using a 1,000 µL pipette. The organoids were trypsin-digested (0.25%, Hyclone, Logan, USA) for 5 min and suspended in fresh growth factor-reduced Matrigel (E6909, Sigma, St. Louis, USA) for plating.

### Organoid treatment and collection

To optimize the culture medium for mammary organoids, we assessed the effects of four single growth factors (2.5 nmol/L FGF2, 2.5 nmol/L FGF7, 2.5 nmol/L FGF10, 50 ng/mL EGF, all from Thermo Fisher Scientific, Waltham, USA) on organoid expansion. The organoids (passage 1) were cultured with six media including BM, BM + EGF, BM + FGF2, BM + FGF7, BM + FGF10 and GM at d 0, respectively. The growth area of organoids was acquired using a microscope (Nikon, Tokyo, Japan) at d 2, 4, and 6. One picture in the center of per dome was taken.

The WNT pathway is conserved across species and plays a pivotal role in controlling cell proliferation and development of mammary gland [24, 25]. To investigate whether the cultured organoids respond to the WNT pathway, we introduced two known inhibitors of WNT, IWR-1-endo (2 μmol/L, S7086, Selleck Chemicals, Houston, USA) and IWP2 (2.5 μmol/L, S7085, Selleck Chemicals, Houston, USA) into the culture medium. The culture medium with same amount of dimethyl sulfoxide (DMSO, D2650, Sigma, St. Louis, USA) as control. The growth area of organoids was acquired using a microscope (Nikon, Tokyo, Japan) at d 1, 3, 5, 7, and 9. The organoids were collected at d 9 for the EdU staining.

To investigate the long-term lactation capabilities of the organoids, we initiated incubation with LM at d 4 and extended it up to d 30. A control group was designed in which LM was replaced by BM at d 6 until to d 30. The organoids were collected at d 9, 20, and 30 for the BODIPY staining.

LXR (liver X receptor) and PPARG (peroxisome proliferator-activated receptor gamma), known key transcription factors, can be activated by T0901317 (a ligand of LXR) or rosiglitazone (a ligand of PPARG) to promote milk fat synthesis in the mammary gland [26–29]. To test whether these transcription factors are functional in the lactating organoids, we incubated rosiglitazone (S2556, 50 μmol/L dissolved in DMSO, Selleck Chemicals, Houston, USA) or T0901317 (S7076, 1 nmol/L dissolved in DMSO, Selleck Chemicals, Houston, USA) in the organoids (passage 1) incubated with LM at d 4. The organoids were collected at d 6 for the BODIPY staining.

### Lipid analysis

To further test whether the main milk compositions are secreted vectorially in our culture system, the mammary organoids (passage 1) were plated in domes in 24-well culture plates (one dome per well, 100 µL of undiluted Matrigel per dome). Each well was seeded with 1,000 organoids. After lactation induction by LM for 2 d, the organoids and culture medium were collected at d 6 for lipid analysis. The whole organoids were collected by rinsing with cold cell recovery solution (Yeasen Biotechnology Co., Ltd., Shanghai, China). The other group with the same number of organoids was digested with trypsin (HyClone, Logan, USA) to collect the cell fraction. Total cellular triacylglycerol (TAG) from the whole organoids and cell fraction were extracted according to the GPO-Trinder triglyceride assay kit protocol (Applygen Technologies, Beijing, China) and suspended in a volume equal to the culture medium in which they were grown. The mass of TAG in culture medium, organoids and cell fraction were determined according to the manufacturer's instructions using a micro-titer plate reader (BioTek Instruments, Inc., Winooski, VT, USA).

### Immunostaining

Collected mammary tissues were fixed with 4% PFA overnight and were then embedded by optimal cutting temperature (OCT) compound (Sakura Finetek USA, Inc., Torrance, CA, USA). Organoids were collected and fixed in 4% PFA for 2 h and then were embedded by OCT. Samples were labeled with antibodies and counterstained with 0.5 mg/mL DAPI (Beyotime Biotechnology Co., Ltd., Shanghai, China). Primary antibodies used included anti-KRT18 (Keratin 18, 1:100, HuaBio, Hangzhou, China) and anti-KRT17 (Keratin 17, 1:200, Proteintech, Wuhan, China). Secondary antibodies were sheep anti-rabbit IgG488 or sheep anti-rabbit IgG594 (Proteintech, 1:1,000, Wuhan, China). To assess cell proliferation index in organoids, the EdU (C0071S, Beyotime Biotechnology Co., Ltd., Shanghai, China) staining was performed according to the manufacturer's procedure. To assess the accumulation of the milk fat in the organoids, lipid droplets were stained by BODIPY (790389, Thermo Fisher Scientific, Waltham, USA) according to the manufacturer's procedure. At least 5 images in each dome of organoids were acquired using a confocal microscope (LSM880, Zeiss, Oberkochen, Germany).

### RNA extraction and sequencing analysis

The organoids cultured with GM or LM (*n* = 3 per groups) were collected at d 6 for RNA isolation. Total RNA was extracted from organoid samples using TRIzol (Thermo Fisher Scientific, Waltham, USA) following the manufacturer’s instructions. The RNA samples (RNA integrity > 8) were used in the subsequent bulk RNA sequencing and quantitative real-time PCR (RT-qPCR). Six sequencing libraries were constructed using the TruSeq RNA Library Prep Kit v2 (Illumina, San Diego, USA), and sequencing was performed on a HiSeq 2500 platform (Illumina, San Diego, USA) by Novogene Co., Ltd. (Tianjing, China). The primary assembly of the Saanen dairy goat genome (NCBI No.: GCA_026652205.1) was used for the sequence alignment through HISAT2 software [30]. Differential expression analysis was then performed using DESeq2 software with |log_2_foldchange|≥ 1 and *P*_adj_ < 0.05. The differentially expressed genes (DEGs) were analyzed and displayed using volcano plots and heat maps. The enrichment of the DEGs between GM and LM groups was examined using Gene Ontology (GO) using KOBAS Knowledgebase (http://bioinfo.org/kobas). To determine the extent to which these organoids could effectively mimic lactating mammary tissue, a comparison for the bulk RNA-seq data was made regarding the changes in transcript levels of genes associated with fatty acid synthesis and milk protein synthesis between organoids (LM group and GM group) and goat mammary tissue (dry-off goat and lactating goat, NCBI accession No. PRJNA637690) [31]. The data of identified sequences from organoids were deposited in the NCBI Sequence Read Archive (SRA) under the accession No. PRJNA1103393.

### RT-qPCR

Synthesis of cDNA was conducted using the PrimeScript TM^RT^ Reagent Kit with gDNA Eraser (Takara Bio Inc., Otsu, Japan) according to the manufacturer’s instructions. The RT-qPCR was performed using SYBR Green (SYBR^®^ Premix Ex Taq™ II, Perfect Real Time, Takara Bio Inc., Otsu, Japan) according to the manufacturer’s instructions. Several genes related to milk fatty acid synthesis [fatty acid synthase (*FASN*), fatty acid binding protein 3 (*FABP3*), and perilipin 2 (*PLIN2*)], six major milk protein [*CSN2*, *CSN3*, casein alpha S1 (*CSN1S1*), casein alpha S2 (*CSN1S2*), and beta-lactoglobulin (*LGB*)] and lactose synthesis [lactalbumin alpha (*LALBA*)] were selected to evaluate the induction lactation of organoids [32–36]. The qPCR reactions were performed in a Bio-Rad CFX96 (Bio-Rad Laboratories Inc., Hercules, USA) using the following conditions: 3 min at 95 °C, 40 cycles of 15 s at 95 °C, and 32 min at 60 °C. All the qPCR data were normalized to ubiquitously expressed transcript (*UXT*). The primer sequences of the genes are previously described [26, 27, 33].

### Proteome profiling

After incubation with LM for 4 d, the mammary organoid samples were collected for protein extraction at d 6. The extracted protein solution was digested with trypsin at 1:50 trypsin-to-protein mass ratio for the first digestion overnight and 1:100 trypsin-to-protein mass ratio for a second 4 h-digestion. For the LC–MS/MS analysis, peptides were separated with a gradient from 6% to 24% solvent B (0.1% formic acid in acetonitrile) over 70 min, 24% to 35% in 14 min and climbing to 80% in 3 min then holding at 80% for the last 3 min, all at a constant flow rate of 450 nL/min on a nanoElute UHPLC system (Bruker Daltonics, Billerica, USA). The peptides were subjected to a capillary source followed by the timsTOF Pro (Bruker Daltonics, Billerica, USA) mass spectrometry. The electrospray voltage applied was 1.60 kV. Precursors and fragments were analyzed at the TOF detector, with a MS/MS scan range from 100 to 1,700 *m/z*. The timsTOF Pro was operated in parallel accumulation serial fragmentation (PASEF) mode. Precursors with charge states 0 to 5 were selected for fragmentation, and 10 PASEF-MS/MS scans were acquired per cycle. The dynamic exclusion was set to 30 s. The resulting MS/MS data were acquired in the data-independent acquisition (DIA) scan mode and processed using MaxQuant search engine (v.1.6.15.0) [28, 29]. Tandem mass spectra were searched against the goat SwissProt database concatenated with reverse decoy database. Trypsin was specified as a cleavage enzyme allowing up to 2 missing cleavages. The mass tolerance for fragment ions was set as 0.02 Da [37]. The mass spectrometry proteomics data have been deposited to the ProteomeXchange Consortium via the PRoteomics IDEntifications (PRIDE) partner repository with the dataset identifier PXD050745 [38].

### Statistical analysis

Treatments for RNA-seq and qPCR were replicated at least 3 times in culture wells, and the qPCR was performed in triplicate. Data of qPCR was analyzed using the 2^−ΔΔCt^ method. The quantification of growth area images was carried out using Image J software (https://imagej.net/ij) by transforming the figures in 8-bit and measuring the area covered by organoids. The percentage growth area of the organoids cultured with different growth factors were normalized to BM group at d 2 while they were normalized to GM group at d 1 in groups cultured with IWR-1-endo or IWP-2. The mean fluorescence intensity for BODIPY staining in organoids was carried out using Image J software. The mean fluorescence intensity was normalized to the number of nuclei per organoid. Detailed information on the statistical test used can be found in the respective Figure legend. All data points shown as technical replicates refer to individual dome of organoids, which are displayed in the respective Figure legend. Prism GraphPad software (San Diego, USA) was utilized for data visualization. Results are expressed as mean ± standard error of the mean (SEM). Statistical differences for the growth area, fluorescence intensity and mass of TAG were determined with a one-way ANOVA (Tukey) using SPSS 19.0 (IBM Corp., Chicago, IL, USA). Significance was declared at *P* < 0.05.

## Results

### Goat mammary organoid isolation and culture

**Fig. 1:**
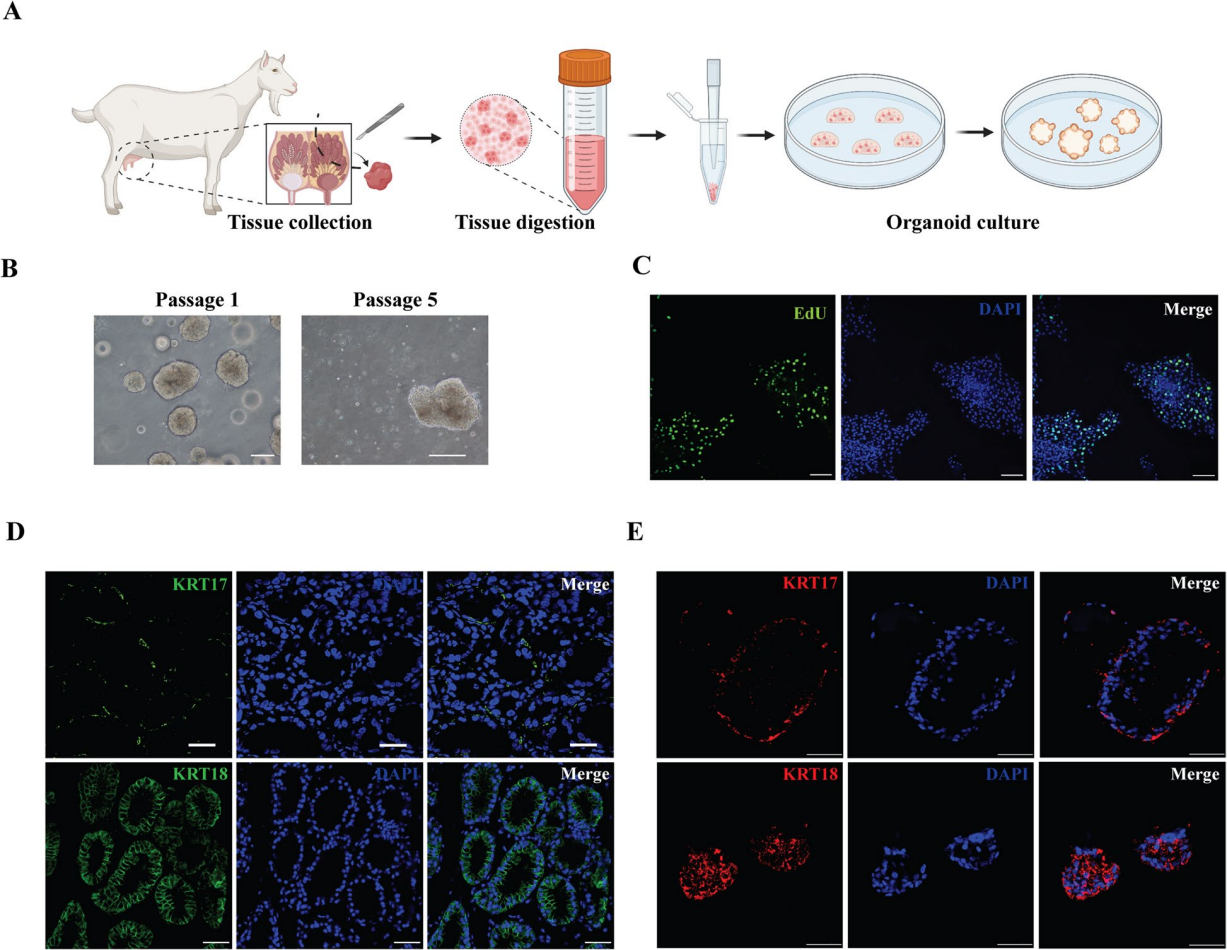
Culture of goat mammary organoids. **A** Schematic diagram illustrating the isolation and culture of goat mammary organoids. **B** Brightfield images showing the normal culture of primary organoids (passage 1) and organoids at passage 5. Scale bar, 200 μm. **C** EdU staining of organoids at passage 5. Scale bar, 100 μm. **D** Immunofluorescence staining of KRT17 and KRT18 in goat mammary tissue. Scale bar, 50 μm. **E** Immunofluorescence staining of KRT17 and KRT18 in goat mammary organoids. Scale bar, 50 μm

The organoid fragments were isolated and cultured within an ECM gel (Fig. 1A). During the growth phase in culture, organoids were typically dissociated and passaged every 2 to 4 weeks to maintain their growth. Bright-field images provided a visual representation of the major organoid phenotypes at passage 1 and 5 (Fig. 1B). To assess the proliferative activity of the cultured organoids, we performed EdU staining, which revealed that even at passage 5, the organoids continued to exhibit high level of proliferation (Fig. 1C). To ensure that the organoids accurately represented the original histological mammary tissue, we conducted immunofluorescent staining using known markers for myoepithelial cells (KRT17) and luminal cells (KRT18) in mammary tissue (Fig. 1D) and organoid sections (Fig. 1E). The comparative immunofluorescence staining demonstrated that the organoids maintained a bilayer structure, consisting of both outer myoepithelial cells and inner luminal cells, which closely resembled the original mammary tissue architecture (Fig. 1D and E).

### Effect of growth factors on the expansion of mammary organoids

**Fig. 2:**
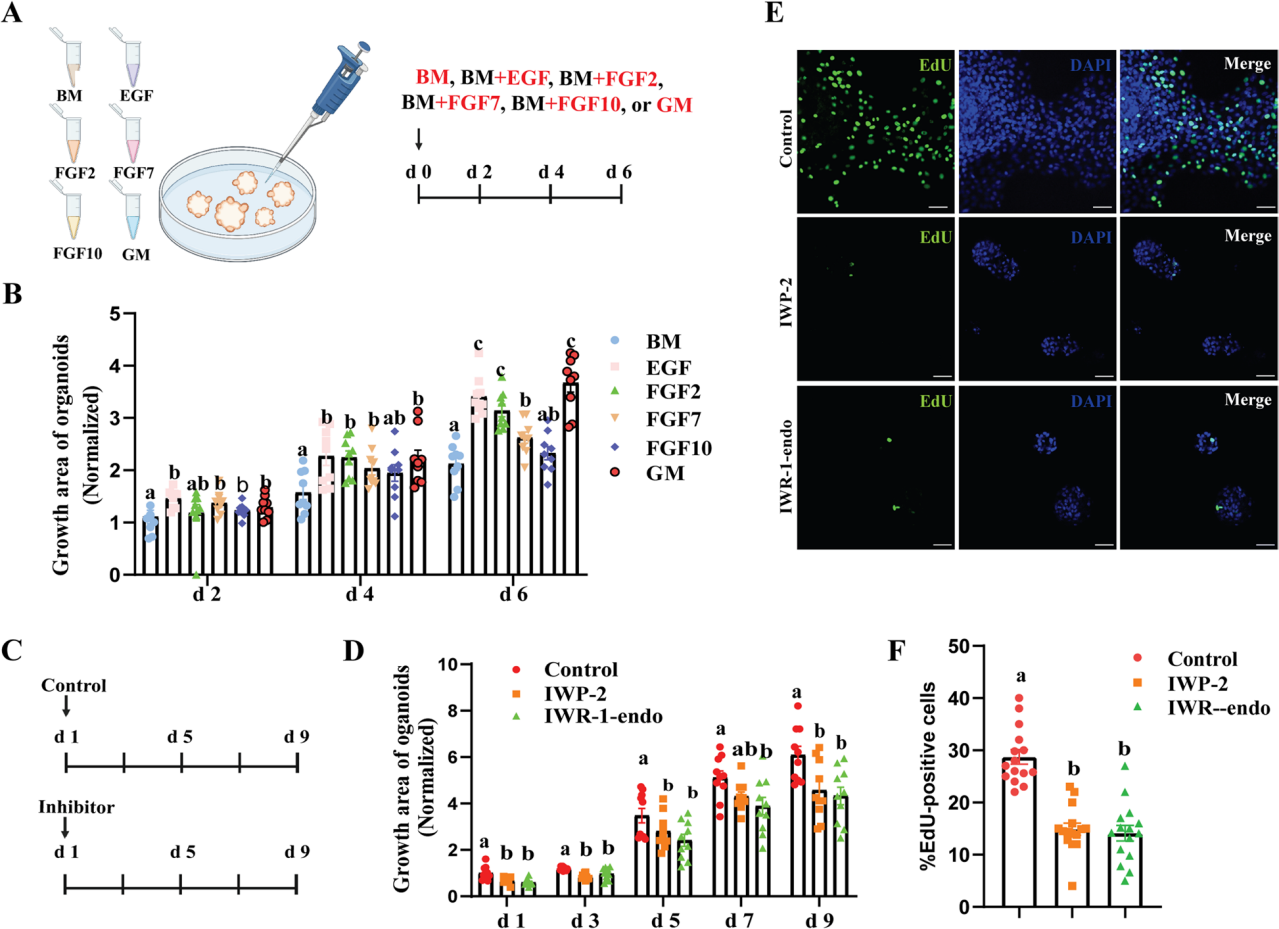
Effects of various growth factors on the growth of goat mammary organoids. **A** Schematic diagram illustrating the effects of various growth factors on organoid growth. **B** Quantification for the growth area of organoids when incubated with various growth factors. Each dot corresponds to one dome of organoids stimulated with various growth factors (*n* = 9 per group). The data are normalized to BM group at d 2. Statistical significance by ANOVA analysis (*P* < 0.05) was indicated by different letters. **C** Schematic diagram illustrating the effects of IWP-2 and IWR-1-endo on organoid growth. The inhibitors were added to culture medium from d 1 until d 9. The medium was changed every two days. **D** Quantification for the growth area of organoids when incubated with control, IWP-2 or IWR-1-endo. Each dot corresponds to one dome of organoids stimulated with control, IWP-2 or IWR-1-endo (*n* = 10 per group). The data are normalized to control group at d 1. **E** Representative images for EdU staining of organoids treated with IWP-2 and IWR-1-endo. Scale bar, 50 μm. **F** Quantification for percentage of the EdU-positive cells in organoids when incubated with IWP-2 and IWR-1-endo. Each dot corresponds to one dome of organoids stimulated with control, IWP-2 or IWR-1-endo (*n* = 10 per group). All data in this figure are presented as mean ± SEM. Statistical significance by ANOVA analysis (*P* < 0.05) was indicated by different letters. BM = basal medium consisting of 5 mg/L insulin, 100 U/mL of penicillin/streptomycin in DMEM/F12. GM = growth medium consisting of BM supplemented with 2.5 nmol/L FGF2, 2.5 nmol/L FGF7, 2.5 nmol/L FGF10, and 50 ng/mL EGF

To optimize the culture medium for goat mammary organoids, we assessed the effects of each four individual growth factors on organoid expansion (Fig. 2A). We observed that EGF, FGF2, and GM significantly promoted the expansion of organoids when compared to the BM group at d 4 and 6 (Fig. 2B and Fig. S1). However, it is noteworthy that extended culture with EGF alone occasionally led to a loss of their 3D organization (data not shown). In this context, GM was chosen for subsequent organoid expansion.

Two inhibitors of WNT pathway were added into GM followed the procedure illustrated in Fig. 2C. Our findings indicate a significant reduction in the growth area of the organoids following the incubation of IWR-1-endo or IWP-2 at d 1, 3, 5, 7, and 9 (Fig. 2D and Fig. S2). This idea is further supported by the observation in a decrease in the ratio of EdU-positive cells when incubated with IWP-2 and IWR-1-endo (Fig. 2E–F).

### Lactation induction in goat mammary organoids

**Fig. 3:**
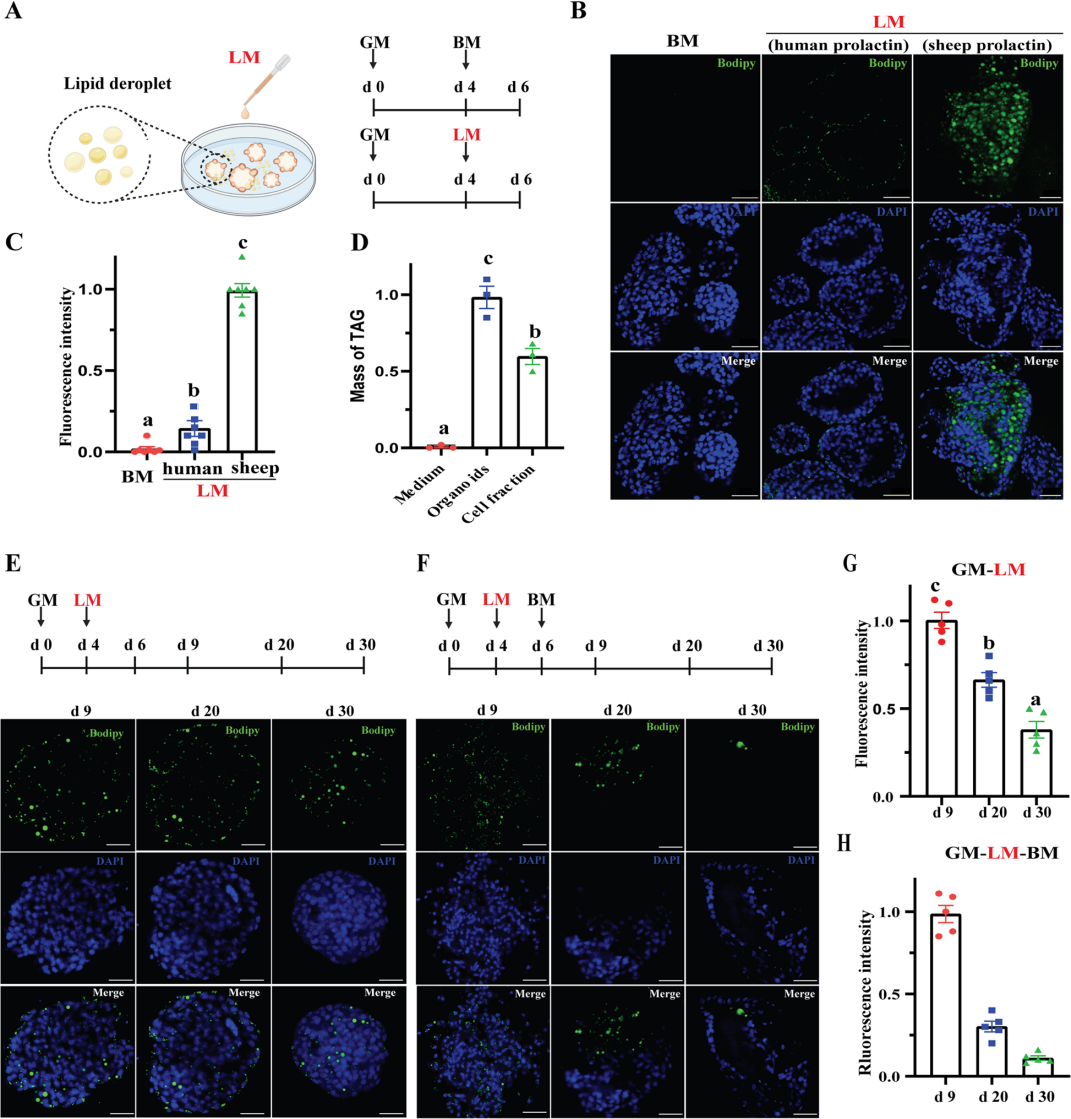
Induced lactation of goat mammary organoids. **A** Schematic diagram illustrating lactation induction in goat organoids by lactogenic medium (LM). **B** Representative images for BODIPY staining of goat organoids after incubation with LM and BM (as control). The LM or BM was incubated at d 4 and the organoids were collected at d 6 for BODIPY staining. Milk fat marked by BODIPY and nuclei marked by DAPI. Scale bar, 50 μm. **C** Quantification of BODIPY staining mean intensity per organoid normalized to the number of nuclei per organoid. Each dot corresponds to one dome of organoids stimulated with BM or LM (*n* = 7 per group). The data are normalized to LM with sheep prolactin. **D** Mass of triacylglycerol (TAG) in the culture medium, whole organoids, and cell fraction of organoids. Each dot corresponds to one individual repeat of organoids stimulated with BM or LM (*n* = 3 per group). Each individual repeat was mixed and analyzed with *n* = 3 domes. The data are normalized to organoid groups. **E**–**F** Schematic diagram and representative images of BODIPY staining for the organoids in extended induction lactation (**E**) and in the involution model (**F**). Milk fat marked by BODIPY and nuclei marked by DAPI. Scale bar, 50 μm. **G**–**H** Quantification of BODIPY staining mean intensity per organoid normalized to the number of nuclei per organoid in (**G**) and (**H**), respectively. Each dot corresponds to one dome of organoids (*n* = 7 per group). The data are normalized to d 9. All the data in this figure are presented as mean ± SEM. Statistical significance with ANOVA analysis (*P* < 0.05) was indicated by different letters. BM = basal medium. GM = growth medium. LM = lactogenic medium

To induce lactation in the mammary organoids, we followed the procedures that the LM was used at d 4 and the organoids were collected at d 6 for BODIPY staining (Fig. 3A). We first assessed the sheep prolactin (2 μg/mL) and human prolactin (2 μg/mL) in GM, respectively. The results from BODIPY staining revealed that sheep prolactin had a more efficient effect on the synthesis of lipid droplets within the goat mammary organoid (Fig. 3B and C) and suggested the successful induction of lactation in the organoids. The different outcomes between prolactin might be due to the high prolactin homology between goats and sheep (100%) followed by human (83%) compared to goats. Based on these data, we selected sheep prolactin (2 μg/mL) as the preferred choice for subsequent induction of lactation in the goat organoids (referred to as LM).

Additionally, BODIPY staining of lactating organoids showed an evidence of fat secretion into the luminal space (Fig. 3B). To confirm this observation, we measured the mass of TAG in culture medium, whole organoids, and cell fraction from organoids, respectively. It was expected to find that there was undetectable TAG in culture medium. It is also observed that the whole organoids had higher level of TAG than cell fraction (Fig. 3D). To investigate the long-term lactation capabilities of the organoids. we initiated incubation with LM at d 4 and extended it up to d 30. BODIPY staining showed that the long-term lactation induction led to a gradual decrease in the content of milk fat within the organoids from d 9 to 30 (Fig. 3E and G). Notably, the removal of LM enhanced the decrease in fat accumulation at d 20 and 30 (Fig. 3F and H).

### Transcript changes in lactating organoids

**Fig. 4:**
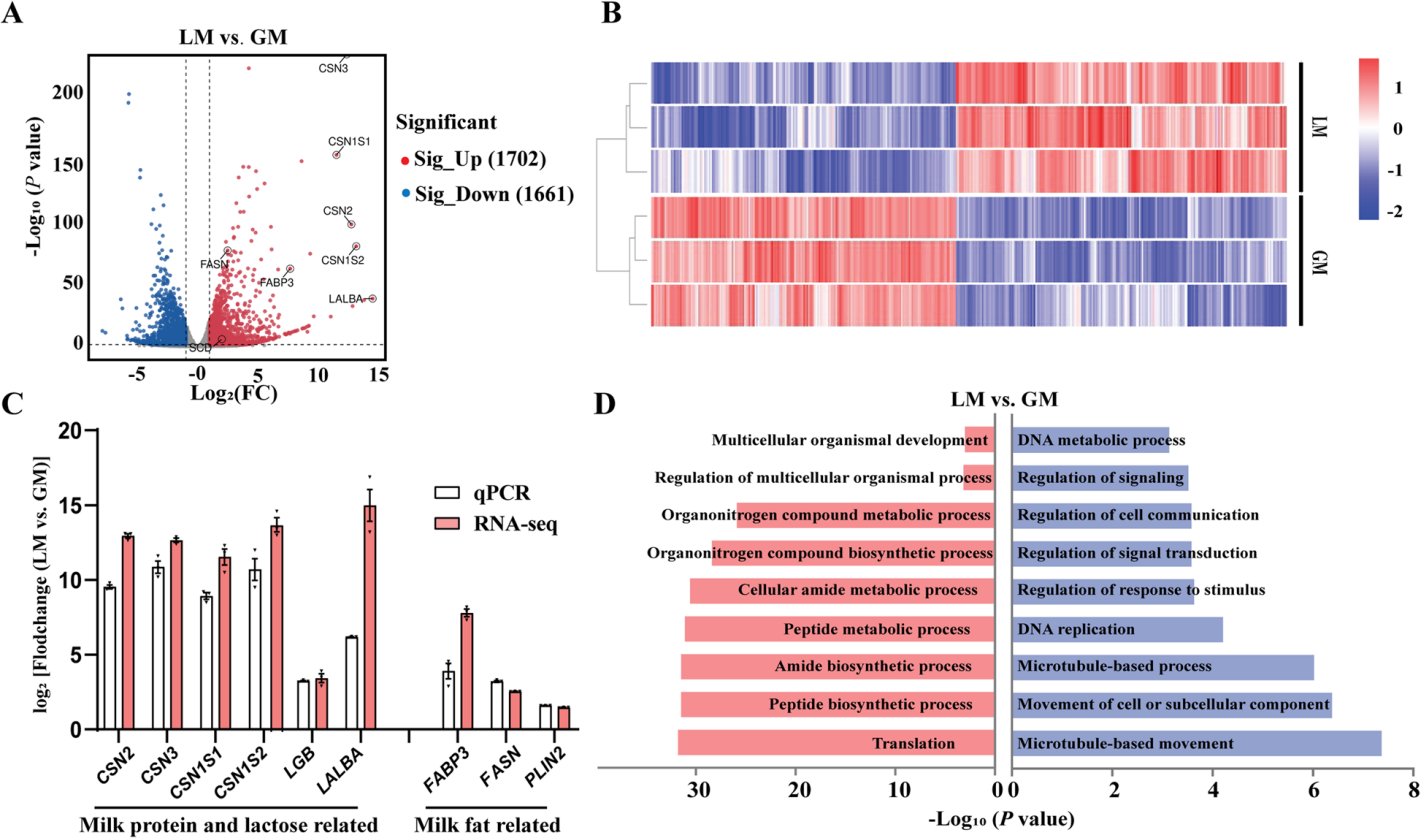
Transcriptome analysis of lactating organoids. **A** Volcano plot of differentially expressed genes between the mammary organoids cultured in lactogenic medium (LM) and growth medium (GM). The genes were selected as *P* value < 0.05 and |log_2_fold change| ≥ 1. **B** Heatmap of differentially expressed genes between LM and GM groups. **C** qPCR verification for selected transcripts and their comparisons with RNA-seq data. These data show the mean of log_2_(fold change) (LM vs. GM) with their SEM. Each dot corresponds to one individual (*n* = 3 per group). The data are presented as mean ± SEM. **D** Pathway enrichment analysis of differentially expressed genes between LM and GM groups. The significance values are displayed as − log_10_(*P* value). GM = growth medium. LM = lactogenic medium

The transcriptomes were compared at d 6 (2 d after stimulation), and showed significant differences in gene expression between the LM and GM organoid groups (Fig. 4A and B). Specifically, we identified 3,363 DEGs (1,702 upregulated genes and 1,661 downregulated genes) in the lactating organoids compared with GM group. To validate the results of RNA sequencing, we performed qPCR to measure the expression of selected nine candidate genes. All the selected genes including *CSN2*, *CSN3*, *CSN1S1*, *CSN1S1*, *LGB*, *LALBA*, *FABP3*, *FASN,* and *PLIN2* were significantly increased in the lactating organoids (Fig. 4A and B). Both RNA-seq and qPCR of these candidate genes showed similar expression patterns (Fig. 4C). These findings verified that the RNA-seq data were reliable and could be further analyzed. The upregulated genes observed in the LM group were primarily enriched in pathways related to metabolism and biosynthesis, such as translation and peptide biosynthesis process (Fig. 4D). On the other hand, the differential expressed genes in the GM group were mainly enriched in pathways related to cell proliferation, such as cell movement and DNA replication (Fig. 4D).

### Milk fat and protein synthesis in lactating organoids

**Fig. 5:**
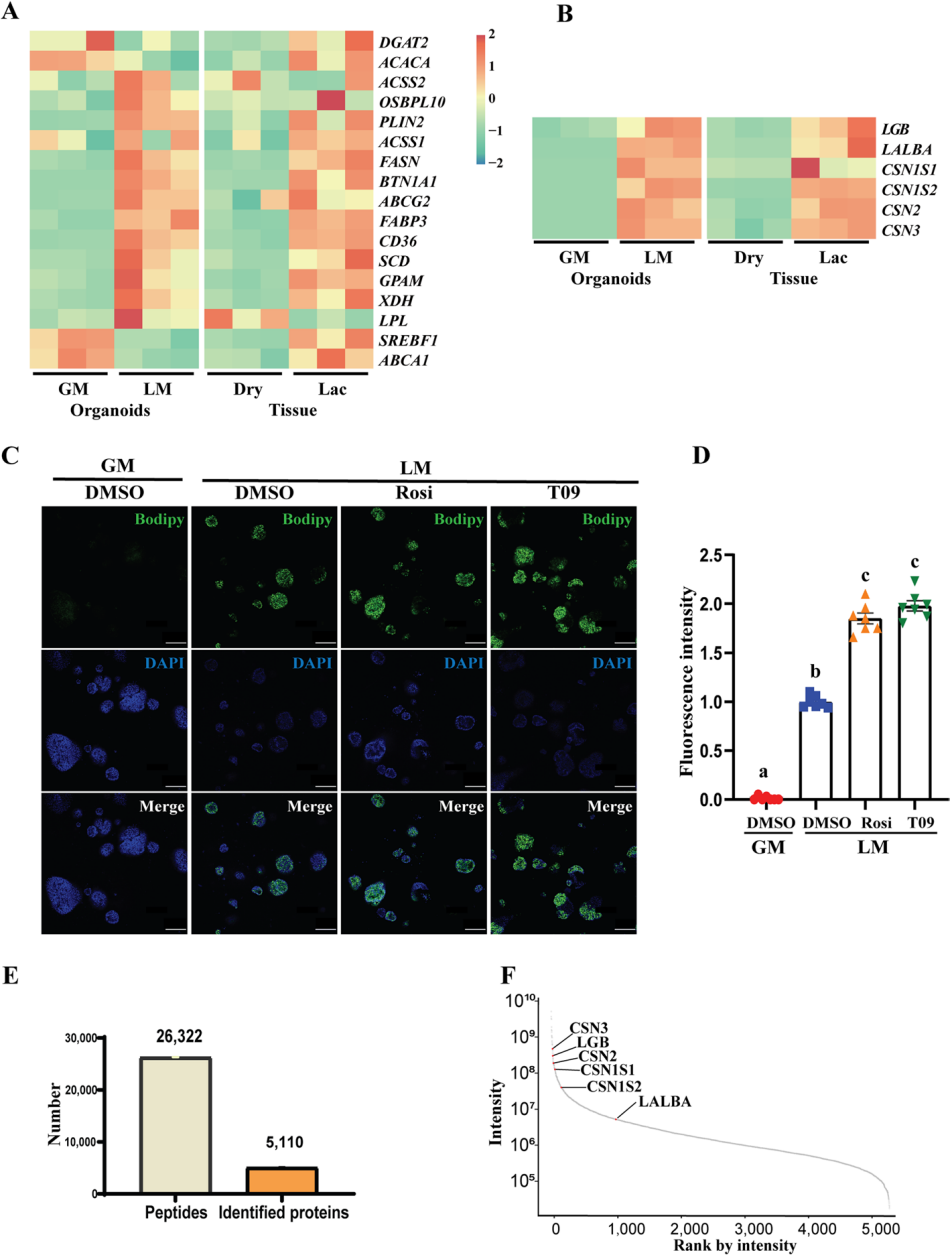
Production of milk fat and protein in the lactating organoids. **A** Heatmap of transcription levels of the lipid metabolism-related genes in organoids cultured in lactogenic medium (LM) and growth medium (GM), and goat mammary tissue at dry (dry) and lactating periods (Lac). **B** Comparison of transcription levels of six genes related to milk protein and lactose synthesis in organoids and goat mammary tissues. (**A**) and (**B**) share a bar value. **C** Representative images for BODIPY staining in the organoids treated with T0901317 (T09) or rosiglitazone (Rosi). **D** Quantification of BODIPY staining mean intensity per organoid normalized to the number of nuclei per organoid in (**C**). Each dot corresponds to one dome of organoids (*n* = 7 per group). The data are normalized to LM with DMSO group. All the data are presented as mean ± SEM. Statistical significance by ANOVA analysis (*P* < 0.05) was indicated by different letters. **E** Identification of proteins in the lactating organoids through proteomic sequencing. **F** Intensity analysis showing the protein expression levels in lactating organoids. The milk proteins are labeled with their symbols

Using the DEGs in the lactating organoids, we extracted the expression of 17 known genes related to fatty acid biosynthesis and lipid droplet formation to assess the efficiency of milk fat production in the lactating organoids [33, 34, 36]. The name of genes and their function descriptions are listed in Table S1 (Additional file 1). The results revealed that in line with the phenotype of lipid droplet accumulation observed in lactating organoids, the key genes associated with fatty acid biosynthesis and lipid droplet formation were significantly upregulated in lactating organoids except for sterol regulatory element binding transcription factor 1 (*SREBF1*), ATP binding cassette subfamily A member 1 (*ABCA1*), acetyl-CoA carboxylase alpha (*ACACA*) and diacylglycerol O-acyltransferase 2 (*DGAT2*) (Fig. 5A). To gain further insights, we compared the changes in transcript levels of these genes in LM group, GM group, dry-off mammary tissue, and lactating mammary tissue. Notably, we found that lactating organoids exhibited a similar gene expression profile as the lactating mammary tissue, with significant upregulation of fatty acid synthesis-related genes (Fig. 5A). Additionally, the expression patterns of six milk protein genes in lactating organoids also closely resembled those observed in lactating mammary tissues (Fig. 5B). Furthermore, the results of BODIPY staining showed a significant increase in lipid accumulation within the organoids following incubation with either T0901317 or rosiglitazone (Fig. 5C and D), suggesting the two key transcription factors (PPARG and LXR) work in the organoids as in the tissue.

To confirm the expression levels of milk proteins in the lactating organoids, we conducted an analysis using proteomics. We identified a total of 26,322 peptides and 5,110 proteins within the lactating organoids (Fig. 5E). The identified peptides corresponding to milk proteins are detailed in the Additional file 2. Abundance intensity analysis further revealed that milk proteins, including CSN2, CSN3, CSN1S1, CSN1S2, LGB, and LALBA, exhibited high expression levels in the lactating organoids (Fig. 5F).

## Discussion

The cultivation of mammary organoids represents an important step in bridging the gap between traditional 2D cell culture and the complexity of mammary tissue [39]. Although mammary organoid culture systems have been previously described in rodent and human models, the concentrations of growth factors used in the culture medium were various, leading to differences in organoid proliferation rates [13, 16, 22, 40]. In the present study, we have observed that the role of FGF2 in promoting the proliferation of mammary organoids aligns with findings in mice [18]. Additionally, we have identified the effectiveness of EGF in promoting the growth of these organoids. However, it is worth noting that in human studies, the use of high concentrations of EGF alone has been reported to cause mammary organoids to lose their 3D organization and gradually sink [22]. However, the current organoid system provides a valuable opportunity to investigate the impact of growth factors on the development of the mammary gland. This notion is further substantiated by our data on the WNT pathway, where we have observed that inhibiting this pathway hinders the proliferation of goat mammary organoids, as demonstrated by EdU incorporation and percentage of growth area.

Prolactin has been identified as a key reproductive hormone responsible for inducing lactation [1]. Our findings in the current study indicated that prolactin obtained from sheep can effectively stimulate lactation in goat mammary organoids, agreeing with observations made in mouse model [18]. It is noteworthy that the mammary gland has the remarkable ability to sustain lactation for extended periods, often exceeding 6–9 months in dairy goats. Consequently, the 3-week lactation capability demonstrated by goat mammary organoids in our study is indeed an exciting and promising opportunity. This extended period of lactation contrasts with previous studies in mice, where prolactin-induced lactation in mammary organoids was limited to approximately two weeks [18].

However, our results unveil an intriguing phenomenon: when exposed to LM for around 26 d, the lactation capability of the organoids diminished significantly. This reduction is accompanied by a notable decrease in the accumulation of lipid droplets within the organoids. This observation may be attributed to the absence of mammary duct-like structures in mammary organoids, which prevents the efficient expulsion of secreted milk, as into the involution process that occurs upon weaning in mammary tissue [3, 41]. The accumulation of milk within the luminal space could potentially exert cellular lipotoxicity through increasing lumen pressure within the mammary organoids, because excessive accumulation of lipid droplets can lead to endoplasmic reticulum stress and apoptosis [42, 43]. In light of these findings, we propose that this organoid model could serve as a valuable tool for studying the involution process of the mammary gland.

Milk fat serves as a crucial energy source to human or animals. In ruminants, about half of the fatty acids in milk fat are synthesized de novo, involving a network of enzymes. These enzymes encoded by *ACACA*, *FASN*, and acetyl-CoA synthetase 2 (*ACSS2*) collaborate to initiate the de novo fatty acid synthesis [36, 44]. Once fatty acids are synthesized, they are intricately assembled into TAG and lipid droplets for eventual secretion into milk [45–47]. The orchestration of these processes heavily relies on the activity of proteins encoded by the glycerol-3-phosphate acyltransferase, mitochondrial (*GPAM*), *PLIN2*, diacylglycerol O-acyltransferase 1 (*DGAT1*), and butyrophilin subfamily 1 member A1 (*BTN1A1*) [36, 48]. Importantly, the higher mRNA expression of these genes agreed with the significant enrichment of lipid droplets observed in lactating goat mammary organoids. The lower expression level of *ACACA*, *ABCA1* and *DGAT2* might result from the lack of their substrates in the LM. Furthermore, our finding that the activation of PPARG and LXRs by agonists significantly promoted the accumulation of lipid droplets within the organoids supports not only the known role of these transcription factors in the mammary gland, but also that the organoids isolated by our protocol could mimic native tissue in milk synthesis.

The bio-manufacturing of milk using mammary cells represents an increasingly active research direction in food production and offers vital technical advance to address the food shortage challenges facing the food industry [49]. Progress has been made in bio-manufacturing research, particularly in the production of protein components within cell-based milk. However, one of the persistent challenges is the inefficient production of milk proteins in cell systems. In the current study, the data obtained through DIA proteomics highlights a promising breakthrough in the efficient production of milk proteins, including CSN2, CSN3, CSN1S1, CSN1S2, and LGB. These findings are particularly encouraging as they suggest a successful and robust production of essential milk proteins by mammary organoids. However, even if there are no efficient antibodies to staining the milk protein in the organoids, our data of fat secretion into the luminal space and that the content of TAG were only measured in organoids and cell fraction but not the medium further suggests the milk compositions including fat, proteins and lactose are secreted vectorially in our culture system. Nonetheless, the data presented in this study provide substantial evidence supporting the potential of mammary organoids.

## Conclusion

Milk synthesis stands as the distinctive hallmark of the lactating mammary glands. In the current study, using dairy goat as a model, we reported a protocol for the establishment of mammary organoids to mimic the development of the mammary gland and induction of lactation. Upon incubation with LM, these mammary organoids faithfully recapitulated the essential characteristics of lactation, manifesting in the production of cell-based milk that was notably rich in both milk fat and protein. These data represent a significant step forward in our understanding of mammary gland biology and hold the promise of unlocking new avenues for cell-based milk production bio-manufacturing in the food industry.

## Supplementary Information

**Additional file 1: Fig. S1**. Brightfield images showing the organoids incubated with WNT inhibitors including IWP-2 and IWR-1-endo. **Fig. S2**. Brightfield images showing the organoids incubated with various growth factors. **Table S1**. Symbols and descriptions of genes related to lipid metabolism, milk protein and lactose synthesis.

**Additional file 2**. Rank for the identified proteins by proteomics.

## Data Availability

All data measured or analyzed during this work are available from the corresponding author upon reasonable request.

## Abbreviations

ACACA: Acetyl-CoA carboxylase
ACSS2: Acetyl-CoA synthetase 2
BM: Basal medium
BTN1A1: Butyrophilin subfamily 1 member A1
CSN2: Casein beta
CSN3: Casein kappa
CSN1S1: Casein alpha S1
CSN1S2: Casein alpha S2
DEG: Differentially expressed genes
DGAT1: Diacylglycerol O-acyltransferase 1
DGAT2: Diacylglycerol O-acyltransferase 2
DIA: Data-independent acquisition
ECM: Extracellular matrix
EGF: Epidermal growth factor
FASN: Fatty acid synthase
FGF2: Fibroblast growth factor 2
FGF7: Fibroblast growth factor 7
FGF10: Fibroblast growth factor 10
GM: Growth medium
GO: Gene ontology
GPAM: Glycerol-3-phosphate acyltransferase, mitochondrial
LALBA: Lactalbumin alpha
LGB: Beta-lactoglobulin
LXR: Liver X receptor
LM: Lactation medium
KRT17: Keratin 17
KRT18: Keratin 18
OCT: Optimal cutting temperature
PFA: Paraformaldehyde solution
PPARG: Peroxisome proliferator activated receptor gamma
PLIN2: Perilipin 2
UXT: Ubiquitously expressed transcript
