# Supplementary material for: Establishment of goat mammary organoid cultures modeling the mammary gland development and lactation
Source: J Anim Sci Biotechnol. 2024 Oct 1;15:124. doi: 10.1186/s40104-024-01084-7 (PMC11443931; doi:10.1186/s40104-024-01084-7)
Supplement: Supplementary file 1 — Additional file 1: Fig. S1. Brightfield images showing the organoids incubated with WNT inhibitors including IWP-2 and IWR-1-endo. Fig. S2. Brightfield images showing the organoids incubated with various growth factors. Table S1. Symbols and descriptions of genes related to lipid metabolism, milk protein and lactose synthesis. [file 40104_2024_1084_MOESM1_ESM.docx]

**Fig.S1**

Brightfield images showing the organoids incubated with WNT inhibitors including IWP-2 and IWR-1-endo. Scale bar, 200 μm. The six medium including BM, BM+EGF, BM+FGF2, BM+FGF7, BM+FGF10 and GM were incubated with organoids (passage 1) at day 0, respectively. The growth area of organoids was acquired using a microscope (Nikon, Tokyo, Japan) at d 2, d 4, and d 6.


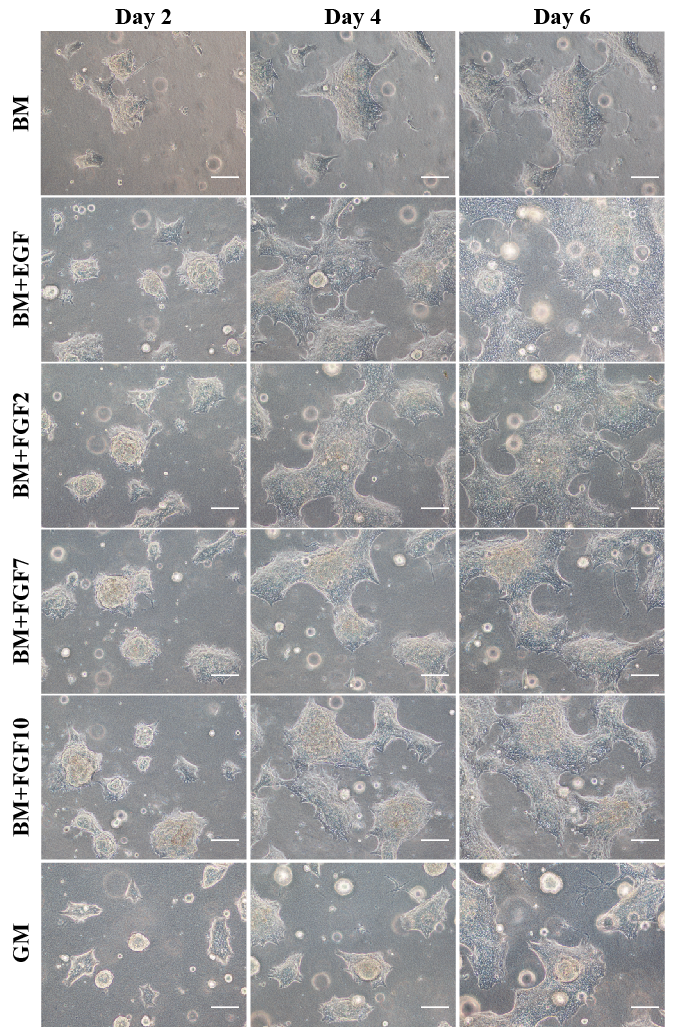


**Fig. S2**

Brightfield images showing the organoids incubated with various growth factors. Scale bar, 200 μm. To investigate whether the cultured organoids respond to the WNT pathway, we introduced two inhibitors of WNT, IWR-1-endo (S7086, Selleck Chemicals, Houston, United States) and IWP2 (S7085, Selleck Chemicals, Houston, United States) into the culture medium. The growth area of organoids was acquired using a microscope (Nikon, Tokyo, Japan) at day 1, 3, 5, 7 and 9.


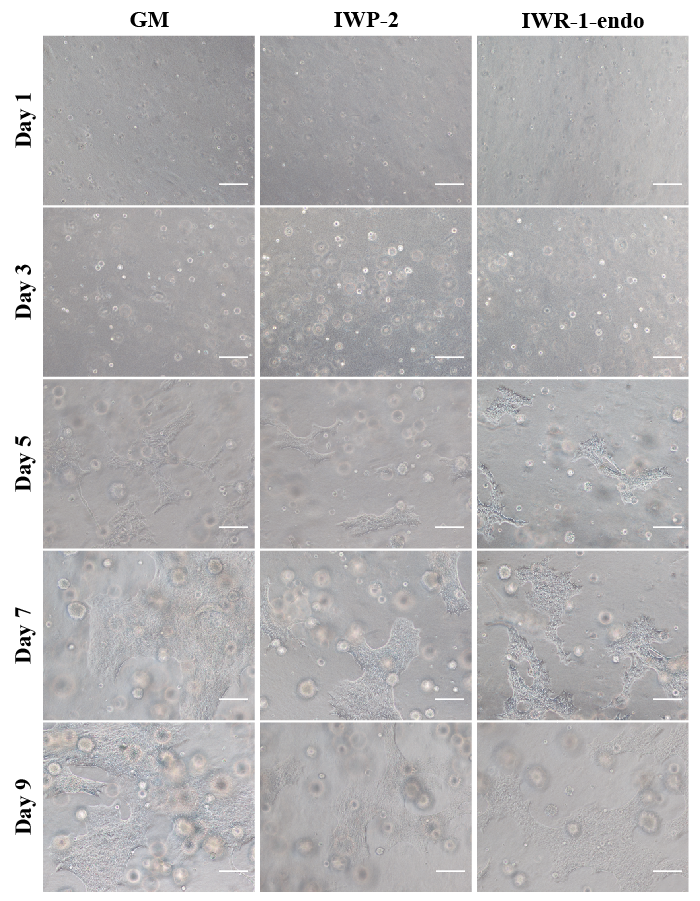


**Table S1**

Symbols and descriptions among genes related to lipid metabolism, milk protein and lactose synthesis investigated.

| Xenobiotic and Cholesterol transport | |
| --- | --- |
| *ABCA1* | ATP-binding cassette, sub-family A (ABC1), member 1 |
| *ABCG2* | ATP-binding cassette, sub-family G (WHITE), member 2 |
| Acetate and FA activation and intra-cellular transport | |
| *ACSS1* | acyl-CoA synthetase short-chain family member 1 |
| *ACSS2* | acyl-CoA synthetase short-chain family member 2 |
| *FABP3* | Fatty acid-binding protein, heart |
| Fatty acid synthesis and desaturation | |
| *ACACA* | Acetyl-coenzyme A carboxylase alpha |
| *FASN* | Fatty acid synthase |
| *SCD* | Stearoyl-CoA desaturase (delta-9-desaturase) |
| Lipid droplet formation | |
| *PLIN2* | Perilipin 2 |
| *BTN1A1* | Butyrophilin, subfamily 1, member A1 |
| *XDH* | Xanthine dehydrogenase |
| FA import into cells | |
| *CD36* | CD36 molecule (thrombospondin receptor) |
| Triacylglycerol synthesis |  |
| *GPAM* | Glycerol-3-phosphate acyltransferase, mitochondrial |
| *DGAT2* | Diacylglycerol acyltransferase 2 |
| Regulation of transcription | |
| *SREBF1* | Sterol regulatory element-binding transcription factor 1 |
| *PPARG* | Peroxisome proliferator-activated receptor gamma |
| *LXR* | Liver X receptor |
| Sphingolipid synthesis |  |
| *OSBPL10* | Oxysterol binding protein-like 10 |
| **Milk protein synthesis** | |
| CNS2 | Casein kappa |
| CNS3 | Casein beta |
| CSN1S1 | Casein alpha S1 |
| CSN1S2 | Cain alpha S2 |
| LGB | Beta lactoglobulin |
| **Lactose synthesis** | |
| *LALBA* | Lactalbumin alpha |
